# Impact of Subharmonic and Aperiodic Laryngeal Dynamics on the Phonatory Process Analyzed in Ex Vivo Rabbit Models

**DOI:** 10.3390/app9091963

**Published:** 2019-05-13

**Authors:** Fabian Thornton, Michael Döllinger, Stefan Kniesburges, David Berry, Christoph Alexiou, Anne Schützenberger

**Affiliations:** 1 Department of Otorhinolaryngology, Division of Phoniatrics and Pediatric Audiology, University Hospital Erlangen, Friedrich-Alexander-University Erlangen-Nürnberg, 91054 Erlangen, Germany; 2 Laryngeal Dynamics Laboratory, Division of Head and Neck Surgery, David Geffen School of Medicine at UCLA, 1000 Veteran Ave, 31-24 Rehab Center, Los Angeles, CA 90095-1794, USA; 3 Section of Experimental Oncology and Nanomedicine (SEON), Department of Otorhinolaryngology, Head and Neck Surgery, Medical School, Else Kröner-Fresenius-Stiftung-Professorship, FAU Erlangen-Nürnberg, 91054 Erlangen, Germany

**Keywords:** ex vivo phonation, rabbit model, aperiodic dynamics, subharmonic dynamics, high-speed digital imaging

## Abstract

Normal voice is characterized by periodic oscillations of the vocal folds. On the other hand, disordered voice dynamics (e.g., subharmonic and aperiodic oscillations) are often associated with voice pathologies and dysphonia. Unfortunately, not all investigations may be conducted on human subjects; hence animal laryngeal studies have been performed for many years to better understand human phonation. The rabbit larynx has been shown to be a potential model of the human larynx. Despite this fact, only a few studies regarding the phonatory parameters of rabbit larynges have been performed. Further, to the best of our knowledge, no ex vivo study has systematically investigated phonatory parameters from high-speed, audio and subglottal pressure data with irregular oscillations. To remedy this, the present study analyzes experiments with sustained phonation in 11 ex vivo rabbit larynges for 51 conditions of disordered vocal fold dynamics. (1) The results of this study support previous findings on non-disordered data, that the stronger the glottal closure insufficiency is during phonation, the worse the phonatory characteristics are; (2) aperiodic oscillations showed worse phonatory results than subharmonic oscillations; (3) in the presence of both types of irregular vibrations, the voice quality (i.e., cepstral peak prominence) of the audio and subglottal signal greatly deteriorated compared to normal/periodic vibrations. In summary, our results suggest that the presence of both types of irregular vibration have a major impact on voice quality and should be considered along with glottal closure measures in medical diagnosis and treatment.

## 1. Introduction

Voice plays an essential role in interpersonal communication. Therefore, healthy voice production is indispensable and pathologies are associated with a significant loss of quality of life. Various professions deal with this subject, including phoniatricians and speech language pathologists, whose tasks include the exploration and treatment of voice, speech and language disorders. This area of responsibility also includes gaining a profound understanding of the phonation mechanism that has been researched for many decades [1,2]. An in-depth understanding of these mechanisms is important to derive new findings about speech system dysfunctions from which new behavioral, medical, and surgical approaches may be developed.

**Figure 1.**
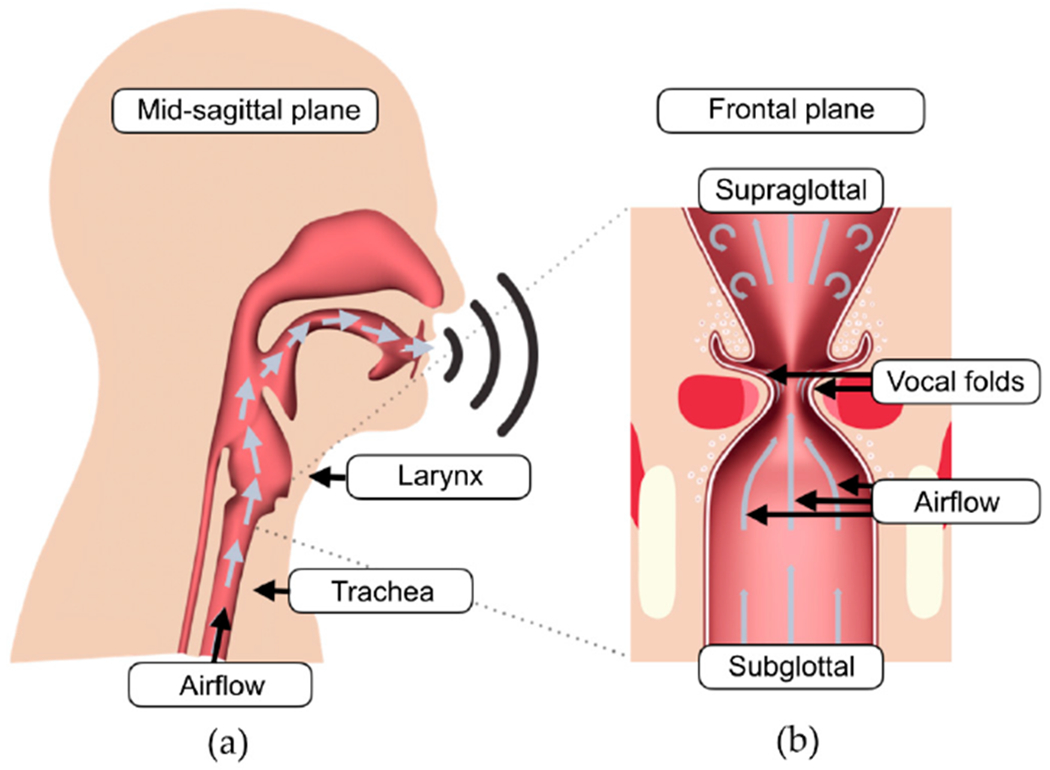
**(a)** Simplified way of air for voice production in overview; **(b)** Schematic longitudinal section through the larynx with laminar airflow under and turbulent airflow above the vocal folds.

The voice originates in the larynx (Figure 1a). Through the core muscles, pressure builds up in the lungs and air is directed through the trachea to the larynx, which causes the vocal folds to oscillate (Figure 1b). By opening and closing of the vocal folds, a fundamental tone is generated (in humans usually between 100–300 Hz for normal phonation [3,4]), which is modulated in the vocal tract and then emitted through the mouth [5].

Many years of research have made it possible to define certain parameters from which it is feasible to draw conclusions about the histological structures, the voice acoustics and the biomechanical components of the vocal folds. These include a wide variety of aerodynamic and acoustic parameters, as well as parameters computed from high-speed digital imaging (HSI) data [6–8].

The technical progress and possibility to record high-speed images of the vocal folds during the high frequency phonatory process was a revolution in clinical phoniatric diagnostics. Since the first analog high-speed recording of human vocal folds in 1940 by Farnsworth [9], technology has evolved steadily. In recent years, the cameras have become smaller and faster and the spatial resolution has improved enormously. Particularly when used in the clinical diagnosis of dysphonia, the HSI technique is superior to stroboscopy [10] and it is impossible to imagine phonatory clinical research today without HSI [10–16]. As long as the frame rate of the camera teaches at least 4000 frames per second, the technique enables one to capture an accurate representatlon of the vibratory cycle of the vocol folds [17]. From these images and computed parameters, conclusions can tie drawn regarding dynamic changes [18] and voice disorders and pathologies [10,12,19–23].

The detected vocal fold vibrations can be subdivided into three classifications [24]. These are type 1 (periodic), type 2 (subharmonic) and type it (aperiodic or chaotic) vibrations. Since healthy voice production is assumed to show periodic vibrations [25], the type 1 vibrations in this study are also celled normal or non-disordered vibrations. The type 2 and type 3 vibrations are called irregular or disordered vibrations and are often related to voice pathologies [26]. These three types of vibrations have been systematically investigated in various studies over the years [27–29]. However, not all data can be collected easily in everyday clinical practice from humans. In particular, subglottal measurements cannot (or only under severe conditions) be obtained in vivo; ex vivo experiments on larynges are necessary [30].

Voice production in humans and mammals is very similar [31]; therefore, animal laryngeal studies have been carried out from which conclusions are drawn regarding human physiology. In the literature, voice production of animals and the comparison with humans has already been examined many times. Sheep, canines [32,33], porcines [34,35], and rabbits [36] are particularly suitable for comparison with humans. The first three are used, especially because of the similar laryngeal dimensions to humans, whereas the comparison with rabbit larynges is suitable, because the tissue properties are similar to those of the human larynx [36,37]. Despite this fact, only a few studies of rabbit phonation have been performed. Maytag et al. (2013) [38] suggested a method for reliable extraction of phonatory, acoustic and videokymographic data of ex vivo rabbit larynges, as this form of data collection from ex vivo rabbit larynges was not yet sufficiently investigated. They found that the rabbit data was similar in intralaryngeal variability to canine laryngeal data. In a study published in 2017, Mills et al. [7] made adaptations to an excised booth, primarily used for canine larynges, to examine the phonatory range of ex vivo rabbit larynges. It was found that increasing airflow and elongation affected subglottal pressure, fundamental frequency, sound pressure level (SPL) and the vibratory amplitude. In 2018, Döllinger et al. [39] performed the first systematic study on ex vivo rabbit larynges, analyzing HSI, audio and subglottal pressure data and the influence of glottal gap characteristics on the phonatory process, as such comprehensive analyses were missing in the literature. Significant influences of applied airflow and the vocal fold elongation level on vocal fold closure insufficiency were detected [40,41]. In the study of Döllinger et al. [39], only the periodic type 1 vibration data was analyzed. In the context of data collection, however, additional subharmonic type 2, as well as aperiodic type 3 vibration data were observed and recorded. This subharmonic type 2 and aperiodic type 3 vibration data are subject to systematic investigation in the present work. To the best of our knowledge, no ex vivo study has been previously reported that systematically investigated phonatory parameters from high-speed, audio and subglottal pressure data for disordered (subharmonic type 2 and aperiodic type 3) vocal fold oscillations. Therefore, the aim of this study is to close existing research gaps by (1) investigating the impact of glottis closure insufficiency on phonatory parameters also in the range of type 2 and type 3 vibrations; (2) compare the results with those of Döllinger et al. [39] who reported on periodic type 1 oscillations; (3) compare which of both vocal fold dynamics (subharmonic type 2 or aperiodic type 3) result in worse parameter values; (4) investigate whether the regularity (subharmonic type 2, aperiodic type 3 oscillations) or the glottis closure has a stronger impact on the fluid-structure-acoustic interaction (i.e., interaction of airflow—vocal folds—resulting sound) of the phonatory process.

## 2. Materials and Methods

**Figure 2.**
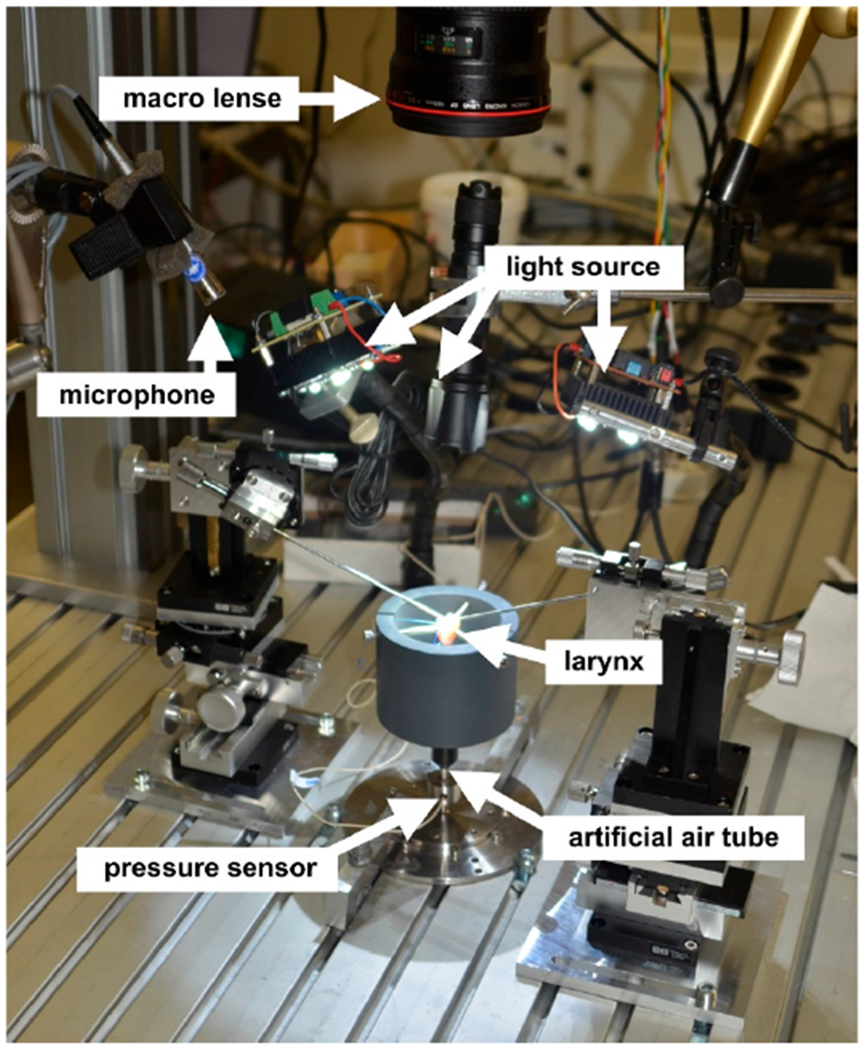
Overview of the experimental setup with inscriptions.

The data acquisition was already previously described in detail [39]; hence the following is only a short overview of the experimental setup and data collection. For detailed information, we refer to the reference study on type 1 oscillations [39]. Data from 11 ex vivo rabbit larynges (New Zealand White, 4–5 kg body weight, ages 14–118 weeks) were used for this study. Since the rabbits were already sacrificed for another study with buprenorphine, no further approval of the ethics committee was required. Ethical acceptability was approved for the previous study (approval number 54-2532.1-54/12). In preparation for the experiments, the larynges were harvested from the sacrificed rabbits. Then the tissue just above and 30 mm below the larynges was surgically removed. To preserve the tissue characteristics, the prepared larynges were quickly frozen at −150 °C in liquid nitrogen and then stored at −80 °C [42]. Before the experiments, the larynges were slowly thawed at 6 °C in a refrigerator. They were then fixated on a 4 mm inside diameter stainless steel tube, which functioned as an artificial trachea. For fixation, a stainless adjustable steel ring was used to prevent air leakage. Rods and positioning screws were used to maintain stable larynx positions, see Figure 1 in [39]. At a distance of 100 mm below the larynx, a hole was drilled in the stainless-steel tube and the pressure sensor was placed there to measure the subglottal pressure. Figure 2 shows the experimental setup.

The subglottal pressure data were measured with an XCS-93-5PSISG pressure sensor (Kulite Semiconductor Products, Inc., Leonia, NJ, USA), which was positioned 100 mm below the larynx on the inside of the stainless-steel tube. The pressure sensor was connected to a PXIe-4330 bridge module (National Instruments, Austin, TX, USA).

The acoustic pressure data was measured using a 4189 1/2” free-field microphone (Brüel & Kjaer, 2850 Nærum, Denmark) mounted above the glottis at a distance of 200 mm and a tilt angle of 45°. The microphone output signal from a Nexus 2690 microphone was then further processed by a PXIe-4492 dynamic signal acquisition module (National Instruments, Austin, TX, USA). Both signals were resolved at 24 bits.

The vocal fold vibrations were recorded by a Phantom V2511 high-speed camera (Vision Research, Wayne, NJ, USA) at 8000 frames per second (fps) with a spatial resolution of 768 × 768 pixels. The videos were recorded at 16 bits. A Canon EF 180 mm 1: 3.5 L USM macro lens (Canon, Ōta, Tokyo, Japan) was used.

The subglottal pressure signal as well as the acoustic signal were synchronously sampled at a rate of f_s_ = 96 kHz. For this purpose, a PXIe-6356 multifunctional data acquisition module (National Instruments, Austin, TX, USA) was used. The setup was controlled using the software LabView (National Instruments, Austin, TX, USA). A detailed description of the setup and its control is given in Birk et al. (2017) [43].

Different levels of vocal fold pre-stress were induced by three different weights applied anteriorly to the thyroid cartilage (w_1_ = 1 g, W_2_ = 2 g, W_3_ = 5 g). The weights were sutured to the thyroid cartilage and used to shift it forward, thereby simulating contraction of the cricothyroid muscle in three different phonatory positions. An MF1 mass flow controller (MKS Instruments, Andover, MA, USA) powered by a PR4000B digital power supply (MKS Instruments, Andover, MA, USA) was used to generate an airflow passing through the artificial stainless-steel trachea and larynx. That air was humified with water vapor from a Neptune Heated Humidifier (Teleflex, Morrisville, NC, USA), and heated to 37 °C to simulate physiological in vivo conditions.

**Figure 3.**
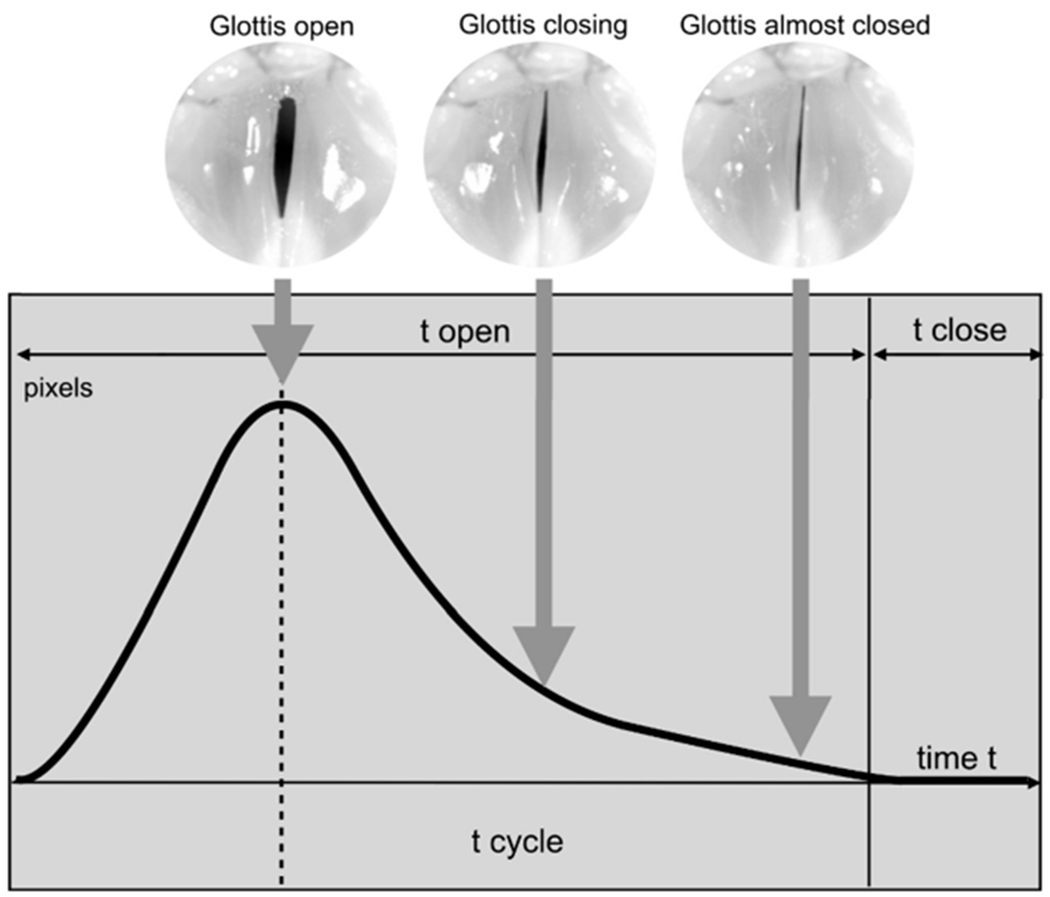
Phonation cycle and typical Glottal-Area-Waveform (GAW) with example images of the glottis during each cycle phase.

The HSI technique allows the computation of quantitative, dynamic-based parameters. Its usefulness has been demonstrated in several previous studies [39,43–46]. The HSI data analysis was performed using the in-house software Glottis-Analysis-Tools (GAT) (University Hospital Erlangen, Erlangen, Germany). The basis for all HSI analyses is the segmentation of the change in the area between the vocal folds over time (i.e., glottis area; given in pixels), which is referred to as the Glottal-Area-Waveform (GAW). A characteristic GAW with the corresponding glottal images is shown in Figure 3. The GAW is used to compute various phonation parameters that describe the phonatory process at the vocal fold level and to obtain information about vocal fold oscillations [47] such as periodicity, glottis closure and left-right symmetry of the vocal folds.

Each experimental run was recorded by the HSI camera for a length of 125ms with sustained phonation (i.e., >42 vibratory cycles, meeting the criteria >20 cycles) as suggested by [48] while the subglottal and audio data were recorded for 500 ms [39].

The computed parameters are subdivided into the groups “GAW parameters”, “Aerodynamic parameters” and “Harmonic measures” according to their data sources; see Table 1.

**Figure 4.**
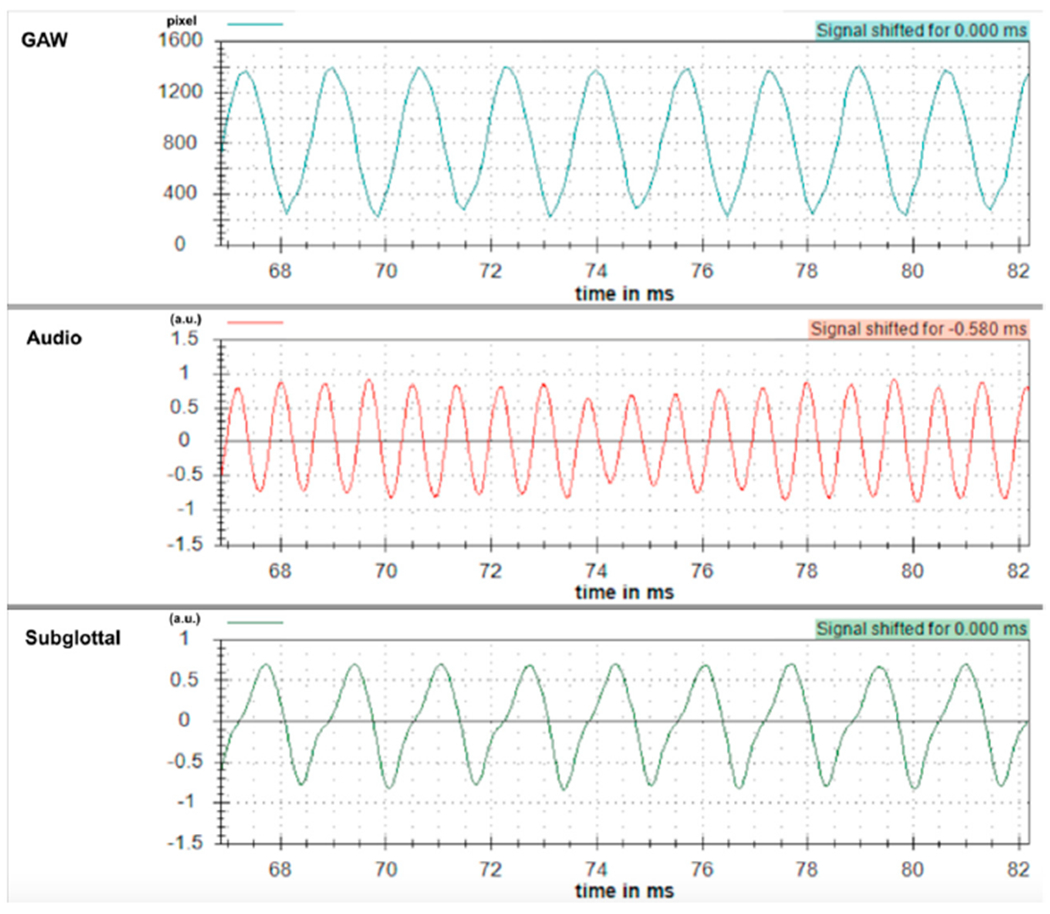
GAW, audio signal, subglottal frequencies in periodic vocal fold vibrations.

**Figure 5.**
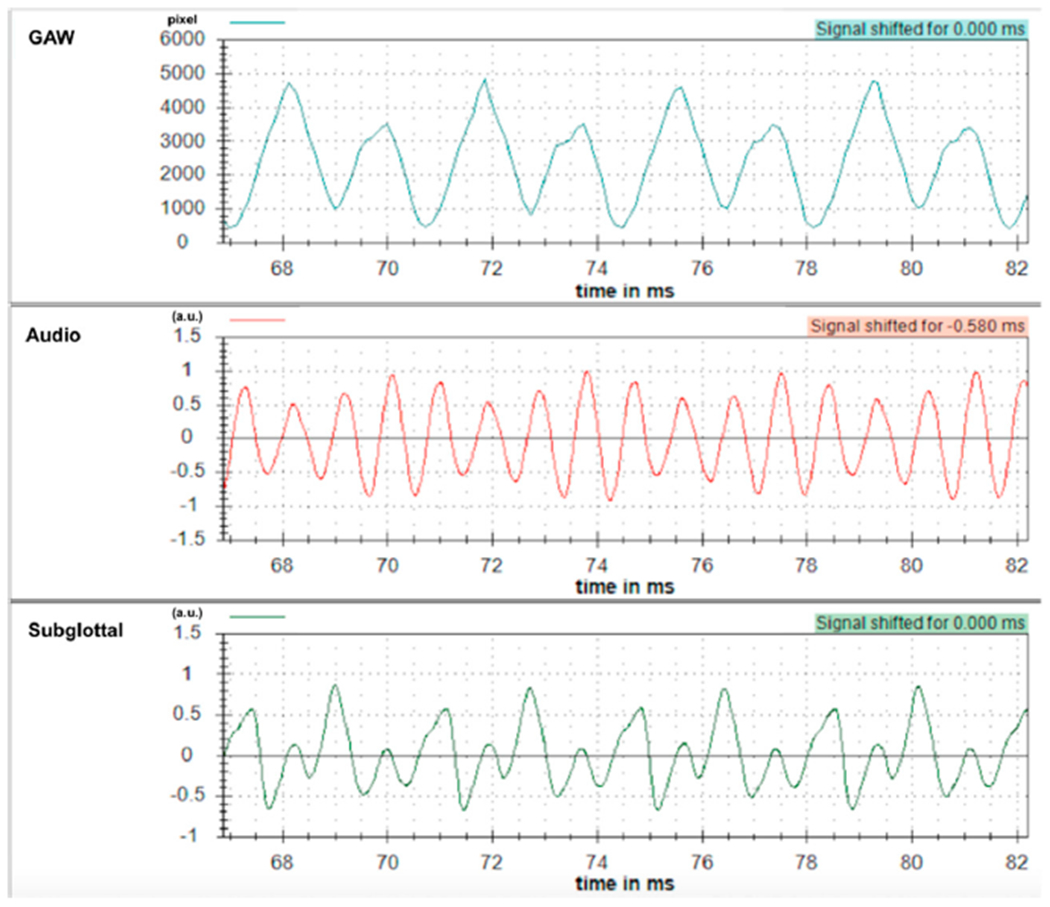
GAW, audio signal, subglottal frequencies in subharmonic vocal fold vibrations with two harmonics in the GAW.

**Figure 6.**
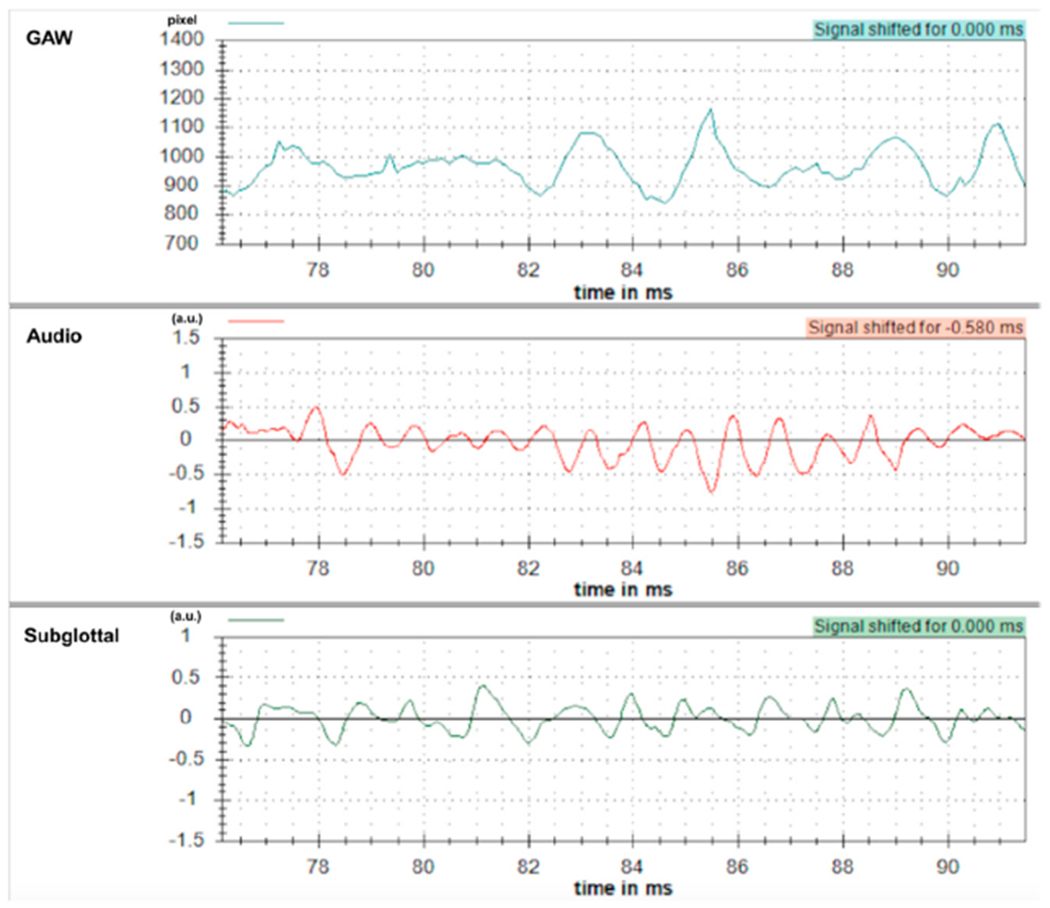
GAW, audio signal, subglottal frequencies in aperiodic vocal fold vibrations i.e., no periodicity recognizable in the GAW.

In the study by Döllinger et al. [39], only periodic vocal fold vibrations containing exactly one harmonic were considered; i.e., these vibrations are considered normal [39]. In contrast, only (1) periodic vibrations with two harmonics and (2) aperiodic vocal fold vibrations are examined in this present study; i.e., these vibrations are considered as disordered. Figures 4–6 show typical examples of the GAW, audio and subglottal frequencies for periodic/normal (Figure 4), subharmonic (Figure 5), and aperiodic vocal fold vibrations (Figure 6). In these figures, the audio signal was shifted by 0.58 ms to correct for the time delay of the acoustic signal; i.e., distance from the vocal folds to the microphone (200 mm) × acoustic wave propagation in air (343 m/s).

Question 1: How does glottal closure insufficiency, expressed by GGI (Table 1), influence the fluid-structure-acoustic interaction within disordered vocal fold dynamics?

The GGI groups were chosen equivalent to Döllinger et al. [39] in order to ensure comparability of the two studies. The three GGI groups are categorized as follows: Best—GGI_1_ ([0; 0.01], desired complete glottis closure during vibration), Medium—GGI_2_ (]0.01, 0.4[, partial closure of the vocal folds), Worst—GGI_3_ ([0.4; 1], no contact of the vocal folds during phonation). Based on the small group sizes, Kruskal-Wallis tests were applied for multiple group comparisons. For subsequent post hoc tests, Mann-Whitney-U tests with a Bonferroni correction (0.05/3 = 0.017) with a resulting significance level of p = 0.017 were used

Question 2: a) Do the subharmonic dynamics influence the phonatory fluid-structure-acoustic interaction process differently compared to aperiodic dynamics? b) Which of both dynamics result in worse phonatory parameter values?

First, the data was examined for the presence of a normal distribution (Shapiro-Wilk). The group differences of the normally distributed data were tested for statistical significance with the parametric t-test, those of the not normally distributed data with the Mann-Whitney-U-test. All statistical analyses were performed using IBM SPSS Statistics 22 software (IBM, Amonk, NY, USA).

## 3. Results

### 3.1. Fundamental Phonatory Parameters

Table 2 provides an overview of the various fundamental phonatory parameters such as fundamental frequency f_0_ (Hz), subglottal pressure P_S_ (Pa), glottal air flow (mL s^−1^), flow resistance R_B_ (Pa s^−1^), and intensity SPL (dB) with the corresponding mean values, minimum and maximum values.

In this study, data of 51 test runs were used. Of these, 35 test runs contained subharmonic (Group_S_) and 16 test runs aperiodic oscillations (Group_A_). As Table 3 shows, the subharmonic and aperiodic oscillations occurred at all airflow intensities (different airflow levels) and elongation levels (different weight levels), but tended to increase with higher air flow and higher elongation.

**Figure 7.**
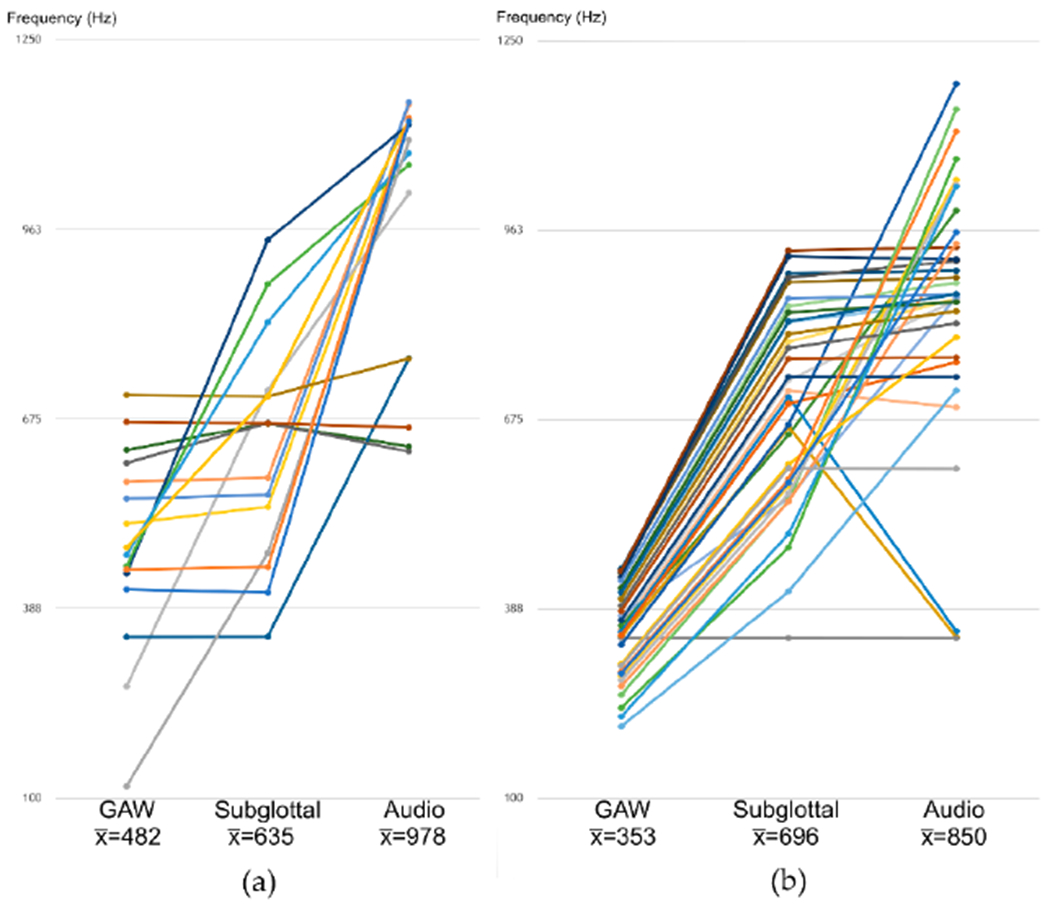
Fundamental frequencies f_0_ subdivided into GAW, subglottal, audio measurements for (**a**) Subharmonic oscillations (**b**) Aperiodic oscillations.

Figure 7a shows the fundamental frequencies of the GAW, subglottal pressure signal and acoustic signal for the subharmonic oscillations. Figure 7b shows this correspondingly for the aperiodic oscillations. The GAW signals show the lowest mean fundamental frequency; see Figure 7a (353 Hz) and Figure 7b (482 Hz). This is followed by the mean fundamental frequency of the subglottal pressure data at 696 Hz (Figure 7a) and 635 Hz (Figure 7b). The highest mean fundamental frequency was found for the audio measurements at 850 Hz (Figure 7a) and 978 Hz (Figure 7b). Despite that increasing tendency, the frequencies show considerable fluctuations; see Figure 7a,b.

### 3.2. Influence, of the Glottal Gap on the Phonatory Process

Question 1: How does glottal closure insufficiency, expressed by GGI, influence the fluid-structure-acoustic interaction within disordered dynamics?

The results of the statistical tests are presented in Table 4. The group differences of all phonation parameters used were tested, which occurred firstly between the three GGI groups and secondly between the two vibration characteristics (Group_S_ and Group_A_). There were 8 out of 13 tests (61.5%) between all three GGI groups and 10 out of 24 post hoc tests (41.7%) that showed statistically significant differences. Statistically significant differences were found in 6 out of the 8 GAW parameters. Most differences in the direct comparison of two GGI groups were found between GGI_1_ and GGI_2_ with 4 statistically significant differences. Between GGI_1_ and GGI_3_, there were 2 statistically significant group differences (Table 4) and 2 of the p-values were slightly above the corrected p-value of 0.017; both with p-values of 0.018. Regarding aerodynamic parameters, the SPL values between the three GGI groups were statistically significantly. For the harmonic measures, CPP_A_ was statistically significantly different between GGI_1_ and GGI_3_.

### 3.3. Influence of the Vibrational Characteristics Subharmonic and Aperiodic on the Phonatory Process

Question 2: a) Do the subharmonic dynamics influence the fluid-structure-acoustic interaction differently compared to aperiodic dynamics? b) Which of both dynamics result in worse parameter values?

The parameters ALR, Stiffness, SPL and CPP_A_ were normally distributed. All other parameters were not normally distributed, so the nonparametric Mann-Whitney-U-test was used for direct group comparison. The corresponding p-values are given in Table 4 in the last column. When comparing the mean values of aperiodic and subharmonic values, statistically significant differences were found in 4 of 8 GAW parameters (ALR, ASQ, CQ, PAI). Of the aerodynamic parameters, only the SPL values were statistically significantly different (p < 0.001). There were no statistically significant differences in the harmonic measures.

### 3.4. Descriptive Statistics

Table 5 shows the means and standard deviations for all parameters of the three GGI groups (GGI_1_ (N = 16), GGI_2_ (N = 29), GGI_3_ (N = 6)). The GAW parameters CQ and OQ increase steadily from GGI_1_ to GGI_3_, while for the parameters ASI and PAI no clear trend is discernible. The remaining parameters decrease from GGI_1_ to GGI_3_. With the exception of P_S_, the values of the other two aerodynamic parameters R_B_ and SPL and those of the harmonic parameters CPP_A_ and CPP_P_ decrease from GGI_1_ to GGI_3_.

The mean and standard deviations of all parameters for the two different vibrational characteristics are given in Table 6. From Group_S_ to Group_A_, all GAW parameters except for ASQ, SQ and PAI decrease; see Table 6. Of the aerodynamic parameters, SPL and P_S_ decrease, while R_B_ increases from Group_S_ to Group_A_. Both CPP_A_ and CPP_P_ decrease from Group_S_ to Group_A_.

## 4. Discussion

A comparison of the computed parameters with other ex vivo rabbit studies has already been reported in Döllinger et al. [39], hence we refer to this work for the interested reader. The main focus of this work is the comparison of periodic type 1 vibrations from [39] with the type 2 and type 3 oscillations in this study, as well as on the comparison within the irregular vibration data.

### 4.1. Fundamental Phonatory Parameters

In comparison with the mean values of Döllinger et al. [39], it is noticeable that the fundamental frequency f_0_ shows higher mean values (+8%) as do the minimum (+9%) and maximum values (+4%); see Table 2. This may be an indication for pathological voice, since Yamauchi et al. [58] found a relationship between incomplete glottis closure and an increase of the fundamental frequency, while investigating data of patients with vocal fold paralysis. Also, similar findings were reported by Wolfe et al. [59] who found a correlation between jitter and the degree of dysphonia by considering the mean fundamental frequency. Hence, an increased fundamental frequency may be seen as an indicator of a pathological or disordered voice [60]. Air flow strength here (120 mL/s) is close to the value of Döllinger et al. [39] of 117 mL/s, which was to be expected since the data were collected during the same series of experiments.

Figure 7 shows the fundamental frequencies (GAW, audio and subglottal). This figure shows that the frequency of the GAW is the lowest and that of the subglottal and audio measurements tend to be higher. However, due to the aperiodicity of the signals, this tendency is not necessarily a causal interrelation. Hence, an interpretation of this effect is difficult.

### 4.2. Phonation Parameters

In comparison to Döllinger et al. [39] not all parameters were computed. The parameters Jitter, Shimmer [61] and Harmonic-To-Noise-Ratio (HNR) [62] are computed in the time domain of the signal and need quasi-periodicity of the signal [63]. Since the aperiodic data show reduced or no periodicity, it was not reasonable to compute and consider these parameters.

#### 4.2.1. GAW Parameters

Eight GAW parameters were considered. The ALR parameter differs statistically significantly between GGI_1_,_3_ as well as between GGI_2_,_3_. Higher ALR values indicate greater deformability and are desirable [39]. In this study, as well as in Döllinger et al. [39]. ALR decreases from GGI_1_ to GGI_3_. Since GGI_3_ has no vocal fold contact, a lower dynamical deformability and consequently a lower value of ALR is plausible compared to GGI_1_ and GGI_2_. It is noticeable that the mean ALR for GGI_3_ from this study is 37% lower compared to the normal ALR at GGI_3_ in [39]; see Table 7. These worse results suggest less deformability for the disordered oscillation data. In comparison of the two vibrational characteristics, the values of the ALR parameters were statistically significantly worse for Group_A_ compared with Group_S_ (Table 4). The difference between the two mean values was −55% (Table 6). Comparing the lowest mean ALR values of GGI (Table 5) and the vibrational characteristics (Table 6), mean ALR was considerably lower for GGI_3_ at a mean value of 4.92, compared with the mean ALR value for Group_A_ (8.14). This suggests that the combination of disordered oscillations (Group_S_ or Group_A_) and GGI_3_ is particularly unfavorable with respect to the oscillatory characteristics of the vocal folds.

The two GAW parameters ASI and PAI, which reflect the dynamic left-right symmetry of the vocal folds, are associated with voice pathologies [58,64]. Surprisingly in this study, as well as in Döllinger et al. [39] the ASI and PAI values had better values for GGI_2_ compared to GGI_1_ and GGI_3_. Regarding the vibrational characteristics, it should be noted that the values both for ASI and PAI showed worse values for Group_A_ compared to Group_S_ (Table 6). The PAI values were statistically significantly worse for the aperiodic vibrations (p = 0.009). This may be an indication that aperiodic oscillations are related to voice pathologies [58,64]. The ASQ values drop for GGI_1,2_ from 0.60 to 0.49 and then increases to 0.52 for GGI_2_3. In comparison, in Döllinger et al. [39], the ASQ values decreased continuously from 0.62 to 0.59 with a glottal gap increase from GGI_1_ to GGI_3_. Unsurprisingly, there are statistically significant differences in the CQ parameter between the GGI groups, because GGI_1_ has a complete glottis closure and GGI_2,3_ show no or only partial glottis closure. The difference in the CQ is also statistically significant between the two vibrational characteristics (Group_S_ and Group_A_). Regarding the GAW data, it is further noticeable that the parameters ASQ, CQ and SQ show strong fluctuations in the mean values of the three GGI groups in comparison to normal data (Table 7). These fluctuations without obvious trends lead to the assumption that these GAW parameters do not seem appropriate to indicate disordered voice.

#### 4.2.2. Aerodynamic Parameters

The energy transfer flow-tissue (R_B_) between the two vibrational characteristics (Group_S_,_A_) increased by 12% to 12363 (Pa s^−1^). In Döllinger et al. [39] R_B_ decreased continuously for increasing glottal gap (GGI_1-3_) by almost 50%. In contrast, here for GGI_1-2_ R_B_ decreased by 30% and then increased by 5% for GGI_2-3_. Compared to the data of Döllinger et al. [39] the value of R_B_ for GGI_3_ in this study was 42% higher; see Table 7. For GGI_1-2_ our findings support other studies that higher energy transfer R_B_ from the glottal flow to vocal folds yields an improved acoustic quality (i.e., higher CPP values) [39,60,65]. Surprisingly, we found contrary results for GGI_2-3_, that R_B_ was increasing/improving while CPP decreased/deteriorated even further; see Table 5. This discrepancy could be due to the fact that none of the other studies were investigating data containing solely disordered oscillations. Further investigations on disordered dynamics are required.

The aerodynamic parameter SPL (dB) differs statistically significantly between the GGI groups, as well as between the two vibrational characteristics (Group_S_, Group_A_); see Table 4. The intensity of the voice signal SPL (dB) decreases from Group_S_ to Group_A_ by 16% from 78.1 dB to 65.9 dB, indicating that aperiodic oscillations (Group_A_) are worse compared to subharmonic oscillations (Group_S_) in terms of efficiency/loudness of voice production.

SPL also decreases from GGI_1_ to GGI_3_ by 22% from 76.7 to 59.5 dB (Table 5). This deterioration of SPL from GGI_1_ to GGI_3_ is stronger than for normal oscillations [39] from 79.1 to 69.4 (−12%). The general decrease in the intensity of the acoustic signal (SPL) may be explained by the fact that with a higher glottal gap index there is a limited oscillatory mobility of the vocal folds as well as an inadequate glottis closure and therefore sound is produced less efficiently.

It is noteworthy that for GGI_3_ 60% higher subglottal pressure than in Döllinger et al. [39] was needed to produce the intensity of 59.5 dB, which is 14% lower than in the data of Döllinger et al. [39]; see Table 7.

Various studies investigated the influence of high subglottal pressure and irregular oscillation (i.e., disordered; Group_S_ and Group_A_) in voice and rough phonation [28,66,67]. We cannot confirm these findings as the overall subglottal pressure regarding the irregular oscillations (Group_S_ and Group_A_) here (Table 2) is 8% lower (1324 Pa) than that of the normal oscillations of Döllinger et al. [39] (1436 Pa).

However, we did find an interrelationship between high subglottal pressure and glottal closure insufficiency. The subglottal pressure P_S_ for GGI_1_,_2_ was relatively similar comparing both studies while PS for GGI_3_ in this study was 60% higher compared to the normal oscillation data [39]; see Table 7. We assume that the combination of strong glottis closure insufficiency (GGI_3_) and disordered voices could be directly linked to high subglottal pressure. Unfortunately, previous studies on phonation data [28,66,67] did not examine the glottis closure insufficiency (i.e., GGI), which is why further investigations are needed to verify this assumption. Since the subglottal pressure data cannot be easily measured in humans, more ex vivo studies are hereby necessary.

#### 4.2.3. Harmonic Measures

The parameter CPP is currently one of the most applied parameters for characterizing dysphonia [68–73]. It is a measure of the voice quality that reflects the degree of occurring harmonics in a voice sample [57,74]. In addition, due to its calculation method, it is suitable for application to aperiodic signals, which is why it is of particular importance in the present study [75].

Just like in Döllinger et al. [39] the values of CPP_A_ and CPP_P_ decreased from GGI_1-3_, and thus became worse (Tables 5 and 7), as the CPP describes the quality of the voice signal [57,68,74,76,77]. Compared to Döllinger et al. [39] it is noticeable that the CPP values for the disordered GGI_1,2_ are about 30% below the normal CPP values for GGI_1,2_, while the disordered CPP value for GGI_3_ is about 45% below the normal CPP values for GGI_3_; see Table 7. Meaning that these deteriorations of CPP for GGI_1-3_ are considerably stronger for the disordered oscillations. These findings apply both to CPP_A_ and CPP_P_. Deteriorations of CPP_A_ and CPP_P_ were also found from Group_S_ to Group_A_, indicating again that aperiodic oscillations (Group_A_) are worse for the acoustic quality compared to subharmonic oscillations (Group_S_). This deterioration was however not as strong as for GGI_1-3_ (Tables 5 and 6).

As Table 8 shows, GGI_3_ is mainly composed of Group_A_. So, the poor CPP_i_ values for GGI_3_ may be based on this over proportional composition. However, since the mean CPP_A_ (15.4 dB) and CPP_P_ (16.0 dB) values of Group_A_ (Table 6) are above the CPP_A_ (11.0 dB) and CPP_P_ (14.0 dB) values of GGI_3_ (Table 5), it is the combination of GGI_3_ and irregularity that seems to be crucial for these poor CPP scores (i.e., reduced harmonics in the acoustic and subglottal signals).

### 4.3. Summary

Only five of the 13 investigated parameters show the same tendencies for both Group_S_ to Group_A_ and GGI_1_ to GGI_3_; see Tables 5 and 6. With increasing glottis closure insufficiency (i.e., GGI_1-3_) nine of the 13 investigated phonatory parameters show similar tendencies compared to the periodic data of Döllinger et al. [39]. The worst mean values of the 4 parameters (ALR = 4.9, SPL = 59.5, CPP_A_ = 11.0, CPP_P_ = 14.0) that are directly linked to voice pathologies, voice disorders and dysphonia such as breathiness and roughness of voice [57,68,74,76,77] were found for GGI_3_. Therefore, we can confirm the findings of Döllinger et al. [39] that the more complete the glottis closure during phonation, the better the acoustic output.

Table 7 shows the percentage change of the (disordered) phonatory parameters compared to (normal/non-disordered) Döllinger et al. [39]. Although GAW and aerodynamic parameters differed compared to [39], no obvious tendency was recognizable. A clear change was observable in the harmonic measures expressed by CPP_A_ and CPP_P_. Both the quality of the subglottal pressure signal (CPP_P_) and of the audio signal (CPP_A_) were noticeably worse compared to [39]. This deterioration (decrease of CPP_A,P_) became stronger with increasing glottis closure insufficiency (i.e., GGI_1-3_), see Table 7.

## 5. Shortcomings

A limiting factor could be the length of time the larynges were frozen. The freezing, storing and thawing was performed according to the study of Chan and Titze [42]. Chan and Titze found that the vocal fold mucosa did not seem to change significantly after 24 h of storage in saline solution at room temperature, nor after one month of frozen storage following quick freezing. Therefore, their findings support the feasibility of using quick freezing to preserve laryngeal tissues for excised larynx experiments, as we performed. However, our larynges were frozen for a maximum of 13 months while the larynges of Chan and Titze were frozen for only one month. This longer time could have an effect on the tissue properties; however, there are no references to this assumption in the literature.

## 6. Conclusions

Since severe deterioration of the subglottal (CPP_P_) and audio (CPP_A_) signal quality occurred regardless which of the two irregular vibration types (subharmonic and aperiodic) were present, we suggest that these two parameters should be considered in medical diagnosis and treatment, alongside with glottal closure characteristics.

As Table 3 shows, disordered oscillations seemed to occur more often under high airflow levels (43%) and high elongation levels (55%). This may suggest that high or over stimulation of laryngeal parameters facilitates disordered phonatory behavior.

To further confirm these assumptions, we suggest further studies investigating disordered laryngeal dynamics with glottis closure insufficiency at different stimulation levels and their impact on the phonatory process, using ex vivo larynx experiments.

## Figures and Tables

**Table 1.** Computed parameters with explanations. ^1^

Abbreviation, Unit, References	Parameter	Meaning and Interpretation
**(A) GAW Parameter**		
GGI (a.u.) [5]	Glottal gap index	Minimum glottal area/maximum glottal area: [0–0.01] glottis entirely closed ]0.01–0.4[ glottis partially closed [0.4–1] little movement and no contact of vocal folds
ALR (a.u.) [49]	Amplitude to length ratio	Dynamic range of GAW (max–min)/glottis length: the larger the more deformable the vocal folds
STIFFNESS (1/frames) [50]		Maximum absolute value of 1st derivative/dynamic range: the higher the value the stiffer the tissue
ASQ (a.u.) [51]	Asymmetry quotient	Speed quotient/(Speed quotient + 1)
CQ (a.u.) [52]	Closing quotient	Glottis closing time/cycle duration 0: glottis does not close at all 0,5: glottis is closed 50% of the cycle duration 1,0: glottis is closed all the time, does not open
OQ (a.u.) [53]	Open quotient	Glottis open time/cycle duration 0: glottis does not open at all 0,5: glottis is open 50% of the cycle duration 1,0: glottis is open all the time, does not close
SQ (a.u.) [53]	Speed quotient	Opening time/closing time
ASI (a.u.) [54]	Amplitude symmetry index	Spatial symmetry of GAW: rate between maximum center and right glottal area, the closer to 1 the more symmetric values are by definition between [0;1]
PAI (a.u.) [55]	Phase asymmetry index	Symmetry in time: deviation in time between center and right GAW amplitude: the closer to 0 the higher the symmetry values are by definition between [0;1]

**(B) Aerodynamic parameters**		
R_B_ (Pa s^−1^) [56]	Laryngeal flow resistance	low-high flow resistance, ratio between the transglottal pressure difference and the mean glottal flow rate. A high flow resistance is desired in phonation
SPL (dB)	Sound pressure level	Intensity of acoustic signal
P_S_ (Pa)	Subglottal pressure	Averaged air pressure measured below the vocal folds

**(C) Harmonic measures**		
CPP_A,P_ (dB) [57]	Cepstral peak prominence	Development of harmonics, the higher the better low: low periodicity of the acoustic signal high: high periodicity of the acoustic signal (computed from the audio “A” and the subglottal pressure “P” signal)

1 Parameters are separated into (A) GAW parameters, (B)/(C) audio, flow and subglottal measurement data.

**Table 2.** Mean values, minimum and maximum values of the fundamental phonatory parameters.

	f_0_ (Hz)	P_S_ (Pa)	Flow (mL s^−1^)	R_B_ (Pa s^−1^)	SPL (dB)
**Mean ± std**	655 ±147	1324 ± 798	120 ± 42	11428 ± 5631	74.3 ± 8.9
**Minimum values**	343	196	42	2587	54.7
**Maximum values**	895	3318	175	21557	90.7

**Table 3.** Frequencies of the test-runs divided into the different airflow levels and elongation levels.

Airflow levels	Σ = 51
1 = onset	3
2–6 = low	15
7–11 = medium	11
12–16 = high	22
Weight levels	Σ = 51

w_1_ = 1 g—low	12
w_2_ = 2 g—medium	11
w_3_ = 5 g—high	28

**Table 4.** Statistical results for the GAW, aerodynamic, and harmonic parameters. ^1^

Parameters	Kruskal-Wallis-test	Post hoc tests (corrected significance level p = 0.017)			Mann-Whitney-U-/t-test

	GGI_1,2,3_	GGI_1,2_	GGI_1,3_	GGI_2,3_	Group_S,A_
**(A) GAW Measures**					
**ALR (a.u.)**	**0.040**	0.776	**0.001**	0.003	**0.000**
**STIFFNESS (frames^−1^)**	**0.014**	**0.014**	0.018	0.300	0.085
**ASQ (a.u.)**	0.054	-	-	-	**0.040**
**CQ (a.u.)**	**0.005**	**0.002**	0.018	0.511	**0.002**
**OQ (a.u.)**	**0.000**	**0.000**	**0.000**	0.124	0.870
**SQ (a.u.)**	0.113	-	-	-	0.187
**ASI (a.u.)**	**0.017**	**0.016**	0.825	0.036	0.429
**PAI (a.u.)**	**0.017**	0.027	0.606	0.020	**0.009**

**(B) Aerodynamic parameters**					
**R_B_ (Pa s^−1^)**	0.038	-	-	-	0.440
**SPL (dB)**	**0.004**	0.981	**0.002**	**0.014**	**0.000**
**P_S_ (Pa)**	0.086	-	-	-	0.715

**(C) Harmonic measures**					
**CPP_A_**	0.020	0.129	0.005	0.066	0.677
**CPP_P_**	0.123	-	-	-	0.231

1 First four columns: Calculated p-values between the three GGI groups. Last column: Calculated p-values for two vibrational characteristics (Group_S_ and Group_A_). Significant p-values are highlighted in bold type.

**Table 5.** Mean values and standard deviations for the GAW, aerodynamic and harmonic measures of the three different GGI groups with tendencies.

Parameters	Mean ± standard deviations			**Tendency for GGI_1-3_**
	GGI_1_	GGI_2_	GGI_3_	
**(A) GAW Measures**				
**ALR (a.u.)**	16.5 ± 4.5	16.1 ± 6.7	4.9 ± 5.1	decrease
**STIFFNESS (frames^−1^)**	0.33 ± 0.06	0.28 ± 0.05	0.26 ± 0.06	decrease
**ASQ (a.u.)**	0.60 ± 0.15	0.49 ± 0.14	0.52 ± 0.05	decrease
**CQ (a.u.)**	0.34 ± 0.14	0.50 ± 0.15	0.48 ± 0.05	increase
**OQ (a.u.)**	0.87 ± 0.13	0.99 ± 0.0	1.00 ± 0.00	increase
**SQ (a.u.)**	2.47 ± 2.50	1.34 ± 0.97	1.25 ± 0.31	decrease
**ASI (a.u.)**	0.74 ± 0.15	0.85 ± 0.08	0.74 ± 0.13	-
**PAI (a.u.)**	0.14 ± 0.10	0.10 ± 0.12	0.14 ± 0.06	-

**(B) Aerodynamic parameters**				
**R_B_ (Pa s^−1^)**	14376 ± 5090	9992 ± 5759	10510 ± 4414	decrease
**SPL (dB)**	76.7 ± 6.5	76.0 ± 7.6	59.5 ± 7.5	decrease
**P_S_ (Pa)**	1783 ± 1073	1050 ± 514	1423 ± 662	-

**(C) Harmonic measures**				
**CPP_A_ (dB)**	17.9 ± 4.3	15.8 ± 6.5	11.0 ± 3.4	decrease
**CPP_P_ (dB)**	19.4 ± 5.8	16.9 ± 5.6	14.0 ± 2.9	decrease

**Table 6.** Mean values and standard deviations for the GAW, aerodynamic and harmonic parameters of the two different vibrational characteristics with tendencies.

Parameter	Mean standard deviation		Tendency for Group_S,A_
	Group_S_	Group_A_	
**(A) GAW measures**			
**ALR (a.u.)**	18.0 ± 5.5	8.1 ± 4.4	decrease
**STIFFNESS (frames^−1^)**	0.30 ± 0.06	0.28 ± 0.05	decrease
**ASQ (a.u.)**	0.50 ± 0.14	0.59 ± 0.14	increase
**CQ (a.u.)**	0.49 ± 0.15	0.35 ± 0.11	decrease
**OQ (a.u.)**	0.96 ± 0.06	0.93 ± 0.15	decrease
**SQ (a.u.)**	1.46 ± 1.11	2.18 ± 2.40	increase
**ASI (a.u.)**	0.81 ± 0.13	0.79 ± 0.11	decrease
**PAI (a.u.)**	0.11 ± 0.13	0.13 ± 0.05	increase

**(B) Aerodynamic parameters**			
**R_B_ (Pa s^−1^)**	11001 ± 5846	12363 ± 5387	increase
**SPL (dB)**	78.1 ± 6.8	65.9 ± 7.3	decrease
**P_S_ (Pa)**	1370 ± 824	1223 ± 781	decrease

**(C) Harmonic measures**			
**CPP_A_ (dB)**	16.1 ± 6.2	15.4 ± 5.3	decrease
**CPP_P_ (dB)**	17.9 ± 6.0	16.0 ± 4.5	decrease

**Table 7.** Percentage change of this present data to normal data in corresponding GGI groups, Döllinger et al. (2018) [39]. ^1^

Parameters	GGI_1_	GGI_2_	GGI_3_
**(A) GAW Measures**			
**ALR (a.u.)**	+8%	+5%	−37%
**STIFFNESS (frames^−1^)**	−3%	+0%	+0%
**ASQ(a.u.)**	−3%	−20%	−12%
**CQ (a.u.)**	+6%	+32%	+17%
**OQ (a.u.)**	+5%	+0%	+0%
**SQ (a.u.)**	+43%	−22%	−18%
**ASI (a.u.)**	−6%	+2%	+1%
**PAI (a.u.)**	+8%	+0%	+8%

**(B) Aerodynamic parameters**			
**R_B_ (Pa s^−1^)**	−4%	−15%	+42%
**SPL (dB)**	−3%	−0%	−14%
**P_S_ (Pa)**	+6%	−25%	+60%

**(C) Harmonic measures**			
**CPP_A_ (dB)**	−25%	−30%	−43%
**CPP_P_ (dB)**	−29%	−36%	−45%

1 Positive deviation means that values increased; negative deviations that our data decreased compared to Döllinger et al. [39].

**Table 8.** Contingency table with the frequencies of GGII_1-3_ and Group_S,A_.

	GGI_1_	GGI_2_	GGI_3_	Σ
**Group_S_**	13	21	1	35
**Group_A_**	3	8	5	16
Σ	16	29	6	51
